# The Effect of Chitosan on Plant Physiology, Wound Response, and Fruit Quality of Tomato

**DOI:** 10.3390/polym14225006

**Published:** 2022-11-18

**Authors:** Fatima El Amerany, Mohammed Rhazi, Gerd Balcke, Said Wahbi, Abdelilah Meddich, Moha Taourirte, Bettina Hause

**Affiliations:** 1 Department of Cell and Metabolic Biology, Leibniz Institute of Plant Biochemistry (IPB), Weinberg 3, 6120 Halle (Saale), Germany; 2 Interdisciplinary Laboratory in Bio-Resources, Environment and Materials, Department of Biology, Higher Normal School, Cadi Ayyad University, P.O. Box 575, Marrakech 40000, Morocco; 3 Laboratory of Sustainable Development and Health Research, Department of Chemistry, Faculty of Science and Technology of Marrakech, Cadi Ayyad University, P.O. Box 549, Marrakech 40000, Morocco; 4 Laboratory of Agro-Food, Biotechnologies and Valorization of Plant Bioresources, Department of Biology, Faculty of Science Semlalia, Cadi Ayyad University, P.O. Box 2390, Marrakech 40000, Morocco; 5 Centre d’Agrobiotechnologie et Bioingénierie, Unité de Recherche Labellisée CNRST (Centre Agro Biotech-URL-CNRST-05), Faculté des Sciences et Techniques, Université Cadi Ayyad, Marrakech 40000, Morocco

**Keywords:** chitosan, fruit maturation, gas exchange, phytohormones, primary and secondary metabolites, tomato growth

## Abstract

In agriculture, chitosan has become popular as a metabolic enhancer; however, no deep information has been obtained yet regarding its mechanisms on vegetative tissues. This work was conducted to test the impact of chitosan applied at different plant growth stages on plant development, physiology, and response to wounding as well as fruit shape and composition. Five concentrations of chitosan were tested on tomato. The most effective chitosan doses that increased leaf number, leaf area, plant biomass, and stomatal conductance were 0.75 and 1 mg mL^−1^. Chitosan (1 mg mL^−1^) applied as foliar spray increased the levels of jasmonoyl–isoleucine and abscisic acid in wounded roots. The application of this dose at vegetative and flowering stages increased chlorophyll fluorescence (Fv/Fm) values, whereas application at the fruit maturation stage reduced the Fv/Fm values. This decline was positively correlated with fruit shape and negatively correlated with the pH and the content of soluble sugars, lycopene, total flavonoids, and nitrogen in fruits. Moreover, the levels of primary metabolites derived from glycolysis, such as inositol phosphate, lactic acid, and ascorbic acid, increased in response to treatment of plants with 1 mg mL^−1^- chitosan. Thus, chitosan application affects various plant processes by influencing stomata aperture, cell division and expansion, fruit maturation, mineral assimilation, and defense responses.

## 1. Introduction

With the increase in the population around the world, the demand for marine resources has undergone a dramatic increase [1]. Thus, the post-catch seafood losses have seen a significant increase which, therefore, leads to economic and environmental issues around the world. These issues occur not only in the landing and distribution chains but also during the consumption of seafood. In order to reduce the impact of seafood use in the environment, the most effective strategies that are recommended are the appropriate use of captured biomass as well as the valorization of seafood waste by extracting valuable compounds, such as pigments (i.e., carotenoids), polymers (i.e., chitin, chitosan), oils (i.e., polyunsaturated fatty acids), proteins (i.e., collagen), and minerals (i.e., hydroxyapatite), for use in agriculture, the pharmaceutical industry, and other fields [2].

Tomato is one of the predominant crops in the world, serving as part of many dishes because of its richness in antioxidants (i.e., lycopene) and vitamins (i.e., vitamin C) [3]. Globally, it is the 12th most popular agricultural product with a cultivated area extended to about 5 Mio ha and an annual production of more than 180 Mio tonnes [4]. In the coming years, the demand for tomato will increase steadily with the concomitant increase in the world’s population reaching 9 billion by 2050 [5]. Thus, a big challenge of agricultural communities is to produce enough nutritious food with improved physico-chemical characteristics and without any alteration of its quality. Therefore, to increase the yield and to obtain high-quality tomato, many agricultural practices have been applied, such as using pesticides, genetic transformation, grafting, and application of nutrients and biostimulants [6,7,8,9,10,11,12]. Among them and regardless of their benefit, the application of biostimulants is the most suitable approach in terms of effectiveness, environmental protection, and easy use without being restricted by the necessity of genetic similarity. Among the biostimulants that are known for their efficiency, there is chitosan, an amino polysaccharide produced from insect cuticles, fungal cell walls, or seafood shells [13]. This polymer acts as an antimicrobial agent, antitranspirant, elicitor, and promoter of the growth of plants and beneficial microorganisms [14,15,16,17]. Regarding its effect on plant growth, it has been shown that it improves photosynthesis via the enlargement of chloroplasts and the stimulation of the biosynthesis of photosynthetic pigments (chlorophyll a, b, and carotenoids) [18]. It also affects plants in a manner similar to phytohormones, which are involved in the regulation of morphogenesis, growth, and development of plants. Application of chitosan increases the content of indole-3-acetic acid (IAA), which promotes cell division, and activates the expression of a *PIN1* gene that regulates auxin translocation [19,20]. In some cases, chitosan application triggers a negative impact on the plant system. For instance, the application of high doses of chitosan (1 mg mL^−1^) with low molecular weight to arabidopsis, tomato, or barley roots arrested shoot development and altered root cell division, growth, and structure, all possibly due to the overproduction of auxin and the alteration of its transport and signaling [20]. This inhibition may be related not only to the high chitosan dose but also to its low molecular weight and the acid used for its solubilization.

Chitosan’s effects on plant systems, however, vary and depend on its physico-chemical characteristics, such as structure, solubility, composition, acetylation degree, and molecular weight, its dose, its application mode, such as foliar spray, coating seeds, amendment, and soil drenching, as well as on the plant species studied and environmental conditions applied [20,21,22,23,24,25,26,27]. Despite the previously mentioned experimental evidence on the positive effects of chitosan as a plant biostimulant, the impact of its application to shoots on plants’ physiology, fruit size, and fruit composition is still not fully understood. Furthermore, there is no report on whether chitosan application at different plant growth stages, such as vegetative growth, flowering, fruit development, and ripening, might have different effects on biological parameters.

Wounding caused by mechanical damage or herbivore attack can threaten plant survival. Therefore, plants have developed many rapid response mechanisms to relieve this stress, either by increasing the expression of defense-related genes, increasing callose deposition, releasing oligosaccharides, or by producing hydrolytic enzymes, most of them mediated by phytohormones, such as jasmonic acid (JA) and abscisic acid (ABA) [28]. The biosynthesis of JA and ABA is initiated by the release of linolenic acid and the cleavage of C40 carotenoid precursor, respectively [29,30]. These phytohormones accumulate in wounded tissues and also mediate systemic signals to increase the immune response in non-wounded tissues [31,32]. Previous studies reported that application of chitosan accelerates wound healing in potato tubers and rice leaves by increasing the thickness of periderm and inducing the levels of proteinase inhibitors and of JA [33,34]. It is not known, however, whether chitosan impacts only the locally treated tissue (local stimulation) or also the non-treated, distant tissues/organs (systemic stimulation).

This work aimed to elucidate the effects of chitosan application on tomato plants in terms of growth, yield, and fruit features using chitosan isolated from *Parapenaeus longirostris* shells. To avoid the negative effect of chitosan on plants, a low dose of acetic acid was used for chitosan solubilization. In addition, the levels of JA and ABA in shoots and roots have been analyzed to clarify alterations in the wound responses after chitosan application to shoots or roots.

## 2. Materials and Methods

### 2.1. Plant Material and Treatments

Chitosan (deacetylation degree 4–17%; molecular weight 318.53 kDa), produced from shrimp waste (*Parapenaeus longirostris*) according to the method described by El Amerany et al. [23], was dissolved in 0.05% (*v*/*v*) acetic acid to a final concentration of 1 mg mL^−1^. From this, three additional solutions of different concentrations (0.25, 0.50, and 0.75 mg mL^−1^) were prepared. Distilled water served as control and corresponded to 0 mg mL^−1^.

Tomato cv. Campbell 33 seeds were germinated in polystyrene trays containing peat substrate at 28 °C. After one week, seedlings were transplanted into plastic pots (4 kg) filled with sand and peat mixture (2:1 [*v*/*v*]). When plants reached the four-leaf stage, chitosan solutions were applied 6 times on the shoots of identically sized plants every two weeks. 

Every week, leaf number (LN) was counted. At the harvest time (twelve weeks after transplantation), stem height (SH), root length (RL), leaf area (LA), fresh and dry weights of shoots and roots (FWS, DWS, FWR, and DWR), and total fresh weight of fruits were determined. SH and RL were measured from the soil contact point to the tip of shoots or roots, respectively. LA was determined using Mesurim software. Plant biomass (shoots, roots, and stems) was weighed before and after their dryness in an oven at 80 °C for 2 days. Additionally, the diameter of ripened fruits was determined using a vernier caliper. Fruits at the same maturity stage were stored at −80 °C or lyophilized for subsequent analysis.

Tomato plants cv. MicroTom were grown in lecaton (particle size 2–5 mm, Original Lamstedt Ton; FiboExClay, Lamsted, Germany) and fertilized weekly with 10 mL Long Ashton solution. Three-week-old seedlings were divided into two groups: one for foliar spray application, while the other one was treated by soil drenching. As mentioned above, leaves or roots were treated with chitosan every two weeks until petals fell and the fruit stage began (in total for a period of 45 days). After 24 h of the latest treatment, the 4th fully developed leaves of each plant were collected and directly flash frozen in liquid nitrogen. The 5th leaves were wounded by squeezing with forceps. After 30 min, lecaton was removed from the roots, from which one half was directly flash frozen, while the other part was wounded by squeezing with forceps. After 30 min, the wounded samples were frozen and all the samples were stored at −80 °C for hormonal analysis and RNA isolation.

### 2.2. Plant Physiological Parameters

The 5th fully developed leaves (from the apex) of Campbell33 were used to estimate chlorophyll fluorescence (Fv/Fm ratio) and stomatal conductance (gs). The measurements were performed after the 2nd, 6th, and 12th week of chitosan application using a fluorometer (Opti-sciences OSI 30 p) and a leaf porometer (LP1989, Decagon Device, Inc., Washington, DC, USA) [35,36].

### 2.3. Phytohormone Analysis

12-oxo-phytodienoic acid (OPDA), JA, jasmonoyl–isoleucine (JA–Ile), and ABA were extracted according to Balcke et al. [37]. Homogenized leaves or roots (50 mg) were mixed with 200 μL methanol spiked with internal standards ([^2^H_5_]–OPDA, [^2^H_6_]–JA, [^2^H_2_]–JA–Ile, and [^2^H_6_]–ABA) and centrifuged at 12,000 rpm at 0 °C for 5 min. Afterwards, 200 μL of supernatant was collected and diluted 10 times by adding 2% formic acid. Phytohormones were pre-purified by solid-phase extraction (SPE) using a 96-well filtration microplate that contained an adsorbens with a high cation exchange capacity (Chromabond, HR-XC, Macherey, Nagel, Düren, Germany) and then analyzed using UHPLC–MS/MS [37].

### 2.4. Real-Time qPCR

Total RNA was isolated from roots using an RNeasy Plant Mini Kit (Qiagen, Hilden, Germany). DNA contamination was removed using a DNA-freeT_m_ Kit (Thermo Fisher Scientific, Waltham, MA, USA) and cDNA was synthesized using a ProtoScript II First Strand cDNA Synthesis Kit (New England Biolabs GmbH, Frankfurt/M., Germany).

Real-time qPCR was carried out as described by El Amerany et al. [23] using the following primer sets: 5′-TTCTACTTCGGCGATTACGGTC-3′ and 5′-GGTTAAGTACGCTCCCTGAACG-3′ for *Allene Oxide Cyclase* (*SlAOC*), 5′-ACATAATAGGCAAAGTCTCA-3′ and 5′-GTTGAAGAAGAAGAGGAGTT-3′ for *9-cis-epoxicarotenoid dioxygenase* 1 (*SlNCED1*), as well as 5′-ACCACGAAGCTCTCCAGGAG-3′ and 5′-CATTGAACCCAACATTGTCACC-3′ for elongation factor, which was used as a housekeeping gene for calculation of relative transcript levels.

### 2.5. Fruit Physico-Chemical Parameters

Moisture content (MC) was measured using the method described by Chafi et al. [38]. Briefly, ripened fruits were weighed to determine their fresh weight (FW). Then, they were placed in an oven (70 °C) for drying until their weight became constant and weighed to measure their dry weight (DW). MC was calculated using the following formula:

(1) $$\mathrm{MC}\;\left(\%\right)=\frac{\left(\mathrm{FW}-\mathrm{DW}\right)\times 100}{\mathrm{FW}}$$

The hydrogen potential (pH) and the total soluble sugar (Brix) of tomato juice were determined using a pH meter (Hanna instrument) and a refractometer, respectively. 

### 2.6. Metabolomics Analysis 

Using the same procedure as described previously to evaluate fruit development and quality, we applied targeted metabolomics [39]. A total of 145 hydrophilic metabolites that cover essential pathways of the central carbon and energy metabolism were analyzed.

Briefly, 25 mg of freeze-dried fruit were homogenized with 0.9 mL of dichloromethane:ethanol (2:1 [*v*/*v*], −80 °C) and 0.2 mL trifluoroacetate and then centrifuged. Afterwards, the aqueous phase was transferred to a new tube and the remainder of the organic phase was re-extracted with 0.05 mL trifluoroacetate and centrifuged. After centrifugation, the obtained supernatant was combined with the previously collected one, frozen at −80 °C, and used for metabolomics analysis of hydrophilic metabolites later. 

All metabolites were separated using a Waters ACQUITY ultra-high-performance liquid chromatography (UHPLC) system, equipped with a Nucleoshell RP18 column (2.1 × 150 mm, particle size 2.1 µm, Macherey and Nagel, GmbH, Düren, Germany), a binary solvent manager, and an ACQUITY sample manager (Waters GmbH, Eschborn, Germany).

Hydrophilic metabolites of the central carbon and energy metabolism were analyzed on a triple quadrupole (QqQ) mass spectrometer using Multiple Reaction Monitoring (MRM) (QTRAP 6500, AB Sciex GmbH, Darmstadt, Germany), equipped with electrospray ionization mass source (ESI) operating in negative ion mode. 

Data processing and peak identification were achieved using MultiQuant™ (version 3.0), MasterView™ (version 1.2), and PeakView™ (version 2.0) software.

### 2.7. Sugar Content

To determine the levels of glucose, fructose, and sucrose, an enzymatic method was used as described previously [40]. An amount of 25 mg of lyophilized tomato fruits was mixed with 500 µL 80% (*v*/*v*) aqueous ethanol and incubated in a thermomixer at 1000 rpm at 80 °C for 1 h, followed by cooling on ice and centrifugation at 14,000× *g* at 4 °C for 5 min. The supernatant was collected and transferred to a new tube for evaporation to dryness (60–78 °C). The residue was dissolved in 250 µL water and subsequently used for sugar analysis in three technical replicates. Reactions were conducted in a 96-well plate with reaction mixtures each containing 100 µL diluted supernatant (7%) and 0.1 U glucose-6-phosphate dehydrogenase (Merck, Darmstadt, Germany) diluted in 100 µL buffer (100 mM Imidazole/HCl, pH 6.92, 10 mM MgCl_2_, 2 mM ATP, 4 mM NAD). The reaction was performed in a microplate reader (Sunrise) and recorded at 340 nm wavelength for 3 min. Subsequently, 0.1 U hexokinase (Merck) was added to determine glucose level. The contents of fructose and sucrose were determined in the same way using 0.2 U phosphoglucose isomerase (Merck) and 1 U invertase (Merck), respectively. Levels were calculated using glucose, fructose, and sucrose as standards [23].

### 2.8. α-Tocopherol Content

Three ripe fruits from the same plant were pooled as one replicate. A total of 25 mg of freeze-dried fruits were mixed with glass and metal beads and 600 µL methanol (+/−10 mg L^−1^ α-tocopherol as internal standard; Sigma-Aldrich, Burlington, MA, USA) containing ascorbic acid (0.2 mg mL^−1^) and homogenized three times in a bead mill. The homogenized extracts were centrifuged at 11,500× *g* for 3 min at 0 °C and then 500 μL supernatant was transferred to a new tube. Then, the remaining residue was extracted again with 200 μL of the same solvent and both supernatants were mixed and stored at −80 °C for subsequent analysis.

Separation of α-tocopherol was carried out using the UHPLC system as above. Solvents used were 10 mmol L^−1^ ammonium acetate (eluent A) and a mixture of 40% 2-propanol and 60% acetonitrile (eluent B). The flow rate and temperature of the column were 400 μL min^−1^ and 45 °C, respectively, and the following gradient applied: 2 min at 60% B, 19 min at 95% B, and 3 min at 60% B. 

The analysis of α-tocopherol was performed by tandem quadrupole time-of-flight mass spectrometry (Q-TOF-MS) using atmospheric pressure chemical ionization (APCI) operating in positive ion mode, MRM detection, and authentic standards.

### 2.9. Lycopene Content

Lycopene levels were determined according to Sadler et al. [41]. A total of 500 mg of fresh tomato fruits was mixed with 12.5 mL ethanol:hexane solution (4:3, *v*/*v*) for 10 min. Then, 2 mL deionized water was added and the mixture was vortexed for 15 sec and left to stand on ice to phase separation. During the extraction processes, tubes were wrapped in aluminum foil to protect them from light.

The upper phase (hexane layer) was used to measure the absorbance at 502 nm in a UV/visible spectrophotometer (Specord 210 Plus, Analytik Jena, Jena, Germany). The lycopene content was determined using the formula described by Hilares et al. [42].

### 2.10. Total Flavonoid Content

The total flavonoids were determined using the method described by Fu et al. [43], with some modifications. Freeze-dried tomato fruits (500 mg) were mixed with 10 mL 80% (*v*/*v*) methanol, containing 1% formic acid and water at volume ratios of 1:10 to 1:15. The mixture was stirred at 150 rpm for 2 h in darkness and at room temperature. After standing for 5 min, the supernatant was filtered through Whatman No 1 filter paper and stored at −20 °C. Then, 500 µL extract was mixed with 300 µL of 5% (*w*/*v*) sodium nitrite. After 5 min of incubation at room temperature, 300 µL of 10% (*w*/*v*) aluminum nitrate was added. After 6 min of incubation, 2 mL of 1 M sodium hydroxide was added to stop the reaction. The absorbance was determined at 500 nm. Finally, the concentration of total flavonoids was estimated by using rutin as a standard.

### 2.11. Macronutrient Content

Macronutrients in tomato fruits were determined using the method described by Segarra et al. [44]. Briefly, 100 mg of freeze-dried fruits were digested with 2 mL nitric acid, 2 mL deionized water, and 1.2 mL hydrogen peroxide at 90 °C for 72 h. The resulting solution was filtrated and diluted with deionized water. The concentrations of N, P, and K were determined using ICP-AES (Varian-Vista, Mulgrave, Australia).

### 2.12. Statistical Analysis

All data except those of metabolomics were analyzed by CoStat 6.400 (CoHort software) and significant differences between treatments were determined using one-way or two-way ANOVA, followed by Duncan’s test at *p* ≤ 0.05. All biological replicates taken into account originated from one experimental set-up. However, all experiments were repeated and showed similar results. Principal component analyses (PCA) and partial least squares discriminant analysis (PLS-DA) were performed by web application metaboanalyst (https://www.metaboanalyst.ca/ (accessed on 20 September 2022)) to visualize correlations between treatments and variation in the targeted metabolomics study.

## 3. Results

### 3.1. Chitosan Effects on Plant Growth and Physiological Parameters

To study the potential effects of chitosan applied to shoots, the phenotype of tomato plants treated with chitosan was analyzed. For this, plants were treated every two weeks with different doses of chitosan ranging from 0.25 up to 1 mg mL^−1^, described as Ch0.25 up to Ch1. Treatments with water served as “non-treated” controls. 

**Figure 1:**
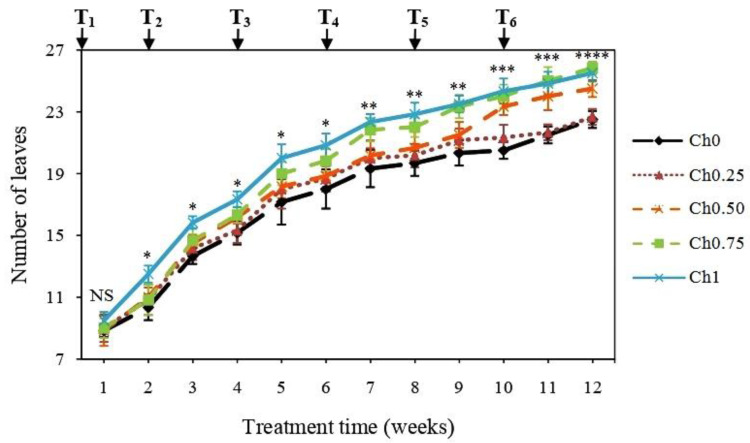
Changes in leaf number of tomato plants receiving different doses of chitosan. Five treatments were applied until fruit ripening with 0 mg mL^−1^ (Ch0), 0.25 mg mL^−1^ (Ch0.25), 0.50 mg mL^−1^ (Ch0.50), 0.75 mg mL^−1^ (Ch0.75), and 1 mg mL^−1^ (Ch1) chitosan. T_1_, T_2_, T_3_, T_4_, T_5_, and T_6_ correspond to the 1st, 2nd, 3rd, 4th, 5th, and 6th application of chitosan. Results were expressed as the mean of 6 independent replicates ± SD. NS, no significant difference between treatments; *, a significant difference between “Ch1” and “Ch0”; **, a significant difference between “Ch1 and Ch0.75” and “Ch0”; ***, a significant difference between “Ch1, Ch0.75 and Ch0.50” and “Ch0”; ****, a significant difference between “Ch1 and Ch0.75”, “Ch0.50”, and “Ch0”.

Determination of LN showed that treatment with Ch1, Ch0.75, and Ch0.50 increased it after the 2nd, 7th, and 10th week of treatment in comparison to non-treated plants (Figure 1), whereas there were no changes in LN after application of a low dose of chitosan (Ch0.25) (Figure 1). 

**Figure 2:**
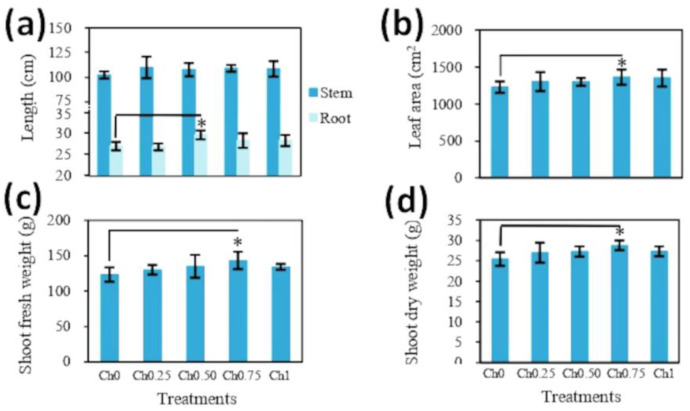
Effect of foliar spray with chitosan on the length of shoots and roots (**a**), leaf area (**b**), and shoot biomass (**c**,**d**) of tomato plants. Five treatments were applied until fruit ripening with 0 mg mL^−1^ (Ch0), 0.25 mg mL^−1^ (Ch0.25), 0.50 mg mL^−1^ (Ch0.50), 0.75 mg mL^−1^ (Ch0.75), and 1 mg mL^−1^ (Ch1) chitosan, and four-month-old plants were analyzed. The results were expressed as the mean of 6 independent replicates ± SD. * indicates significant differences between treatments following one-way ANOVA (Duncan’s multiple range, *p* ≤ 0.05).

Chitosan application to shoots did not affect SH (Figure 2a), but led to alterations in RL, which was only observed in plants treated with medium doses of chitosan in comparison to non-treated plants (Figure 2a). Foliar application of chitosan resulted in a slight increase in LA and fresh and dry weight of the aerial parts of plants (SFW and SDW). Here, the highest values were recorded in plants treated with Ch0.75, showing an increase of around 11%, 16%, and 13% for LA, SFW, and SDW, respectively, compared to control plants (Figure 2b–d). In contrast, chitosan application to shoots had no significant effect on RFW and RDW (Figure S1).

**Figure 3:**
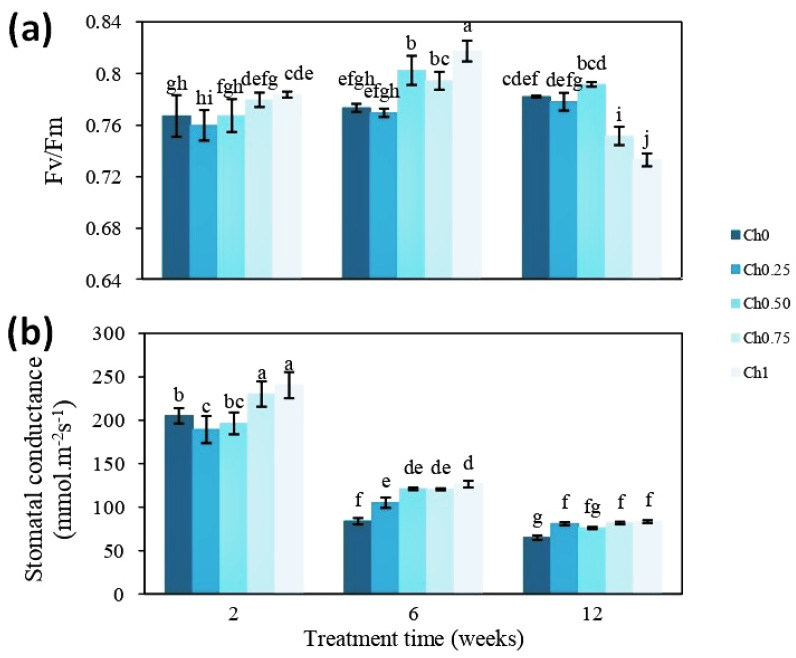
Evolution of chlorophyll fluorescence (**a**) and stomatal conductance (**b**) in plants treated and non-treated with chitosan. Five treatments were applied until fruit ripening with 0 mg mL^−1^ (Ch0), 0.25 mg mL^−1^ (Ch0.25), 0.50 mg mL^−1^ (Ch0.50), 0.75 mg mL^−1^ (Ch0.75), and 1 mg mL^−1^ (Ch1) chitosan. Data were obtained after the 2nd, 6th, and 12th week of treatment. Results were expressed as the mean of 4 independent replicates ± SD. Values followed by different lowercase letters are significantly different according to Duncan’s multiple range test (*p* ≤ 0.05).

To shed light on how chitosan might positively affect shoot growth, two physiological parameters, Fv/Fm ratio and gs, were measured. After 2 weeks of treatment, a significant increase in the Fv/Fm ratio was noted only in plants treated with Ch1 (Figure 3a). The Fv/Fm ratio reached approximately 0.767 for control plants and was increased to 0.784 after treatment with Ch1. The increasing effect was more pronounced after 6 weeks of treatment, where the positive effect of other treatments (Ch0.50 and Ch0.75) was also visible. Here, the Fv/Fm ratio was still higher in plants treated with Ch1 (0.817) (Figure 3a). In plants harboring fruits (12 weeks of treatment), Fv/Fm ratios were, however, decreased after application of Ch0.75 and Ch1 (Figure 3a). Similarly, treatment with chitosan resulted in an increase in gs (Figure 3b). Mainly, the application of higher doses led to a significant increase in gs at all time points, although the general levels were decreasing over the time of the experiment.

### 3.2. Chitosan Effects on Plant’s Response to Wounding

To understand the effect of chitosan on plant defense mechanisms, the levels of JA, its precursor 12-oxo phytodienoic acid (OPDA), and its active form jasmonoyl–isoleucine (JA–Ile) as well as those of ABA, in responses to chitosan applied to shoots or roots as well as to wounding, were determined. In order to prevent accumulation of hormones due to the harvest, plants of the cultivar MicroTom were grown in expanded clay. This enabled the most rapid harvest of shoots and roots.

**Figure 4:**
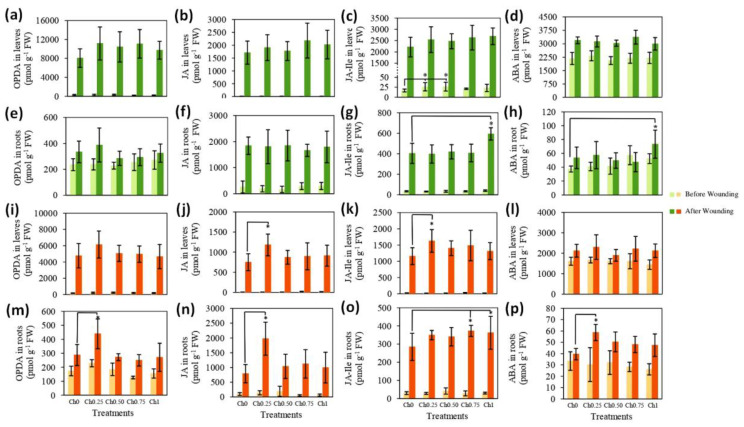
Changes in the levels of plants’ defense hormones after chitosan application to shoots (**a**–**h**) and to roots (**i**–**p**) +/−wounding stress. Means ± SD, *n* = 4. * indicates significant differences between treatments following one-way or two-way ANOVA (Duncan’s multiple range, *p* ≤ 0.05).

Plants treated with different doses of chitosan were either wounded on their leaves or, 30 min later on, roots and samples were taken from leaves and roots before and 30 min after wounding. Quantitative analysis revealed that OPDA, JA, and ABA levels in non-wounded leaves were similar, irrespective of treatment with chitosan (Figure 4a,b,d). The levels of JA–Ile in leaves treated with Ch0.25 and Ch0.50 were, however, approximately 52% higher than those of non-treated plants (Figure 4c). In all plants, wounding of leaves caused a tremendous increase in the levels of OPDA, JA, JA–Ile, and ABA within leaves (Figure 4a–d). Here, no differences were detectable in the hormone levels from leaves treated or non-treated with chitosan (Figure 4a–d).

To explore whether the translocation of phytohormones, produced during wounding of leaves, from the shoots to roots was the reason for their apparent stability in the above-ground part of the plants, we determined the levels of the same phytohormones in roots. As Figure 4e shows, foliar application of chitosan and wounding of roots did not affect the level of OPDA in roots compared to non-treated/non-wounded control. The contents of JA in wounded roots from chitosan-treated and non-treated plants were 540–610% higher than those of non-wounded roots (Figure 4f). The application of Ch1 to shoots resulted in a slight increase in the levels of JA–Ile (18%) in roots and these levels were also significantly higher (47%) after wounding of roots, in comparison to roots of non-treated plants (Figure 4g). As shown in Figure 4h, neither chitosan nor wounding affected the levels of ABA in roots; however, their levels in wounded roots from plants treated with Ch1 were significantly higher than those in control. 

Regarding chitosan application to roots, Figure 4i–p shows no significant changes in phytohormone levels in non-wounded leaves or roots. The contents of OPDA, JA, JA–Ile, and ABA were increased in leaves and roots by wounding. The application of Ch0.25 to roots led, however, to a further increase in the levels of JA and JA–Ile in wounded leaves by 57 and 41%, respectively, and in the level of OPDA, JA, and ABA in wounded roots by 53%, 150%, and 50%, respectively. Additionally, the increase in the content of JA–Ile in wounded roots correlated with the concentration of applied chitosan (Figure 4o).

To confirm the data obtained from the application of chitosan to shoots, the transcript levels of two genes that encode key enzymes of the biosynthesis of OPDA and ABA were determined in roots. Allene oxide cyclase (*AOC*) and 9-cis-epoxycarotenoid dioxygenase (*NCED1*) catalyze the biosynthesis of the correct stereo-isomeric form of OPDA used for JA biosynthesis [45] and the key regulated step in ABA biosynthesis by cleaving xanthophylls into xanthoxin, a precursor of ABA [46], respectively. Table 1 shows that the transcript levels of the *AOC* gene were similar in all root samples, while *NCED1* expression was significantly induced in wounded roots and more pronounced in those from plants treated with Ch1.

### 3.3. Chitosan Effects on Tomato Fruits

Chitosan application significantly increased the total fruit weight by about 50%, 57%, and 36% in plants treated with Ch0.25, Ch0.50, and Ch0.75, respectively. However, there is no significant effect on the yield after the application of Ch1 (Table 2).

Fruit size analysis revealed that Campbell 33 fruits had a flattened shape because the transverse diameter was significantly larger than the longitudinal one and their SI was far from 1 (Table 2). Additionally, fruits from plants treated with chitosan were found to have a slightly flattened shape, except those from plants treated with Ch0.50 appearing in an almost rounded shape with an SI value close to 1 (Table 2). Moreover, the continuous application of Ch1 for 3 months decreased fruit size and water content and increased the total soluble sugars (Brix values, Table 2). Further, the pH values were significantly increased in fruits from plants treated with Ch0.75 and Ch1 compared to non-treated plants (Table 2).

To shed light on how chitosan affects the metabolic pathways of the primary metabolism, targeted profiling for polar metabolites was conducted and the data set was analyzed by multivariate methods.

**Figure 5:**
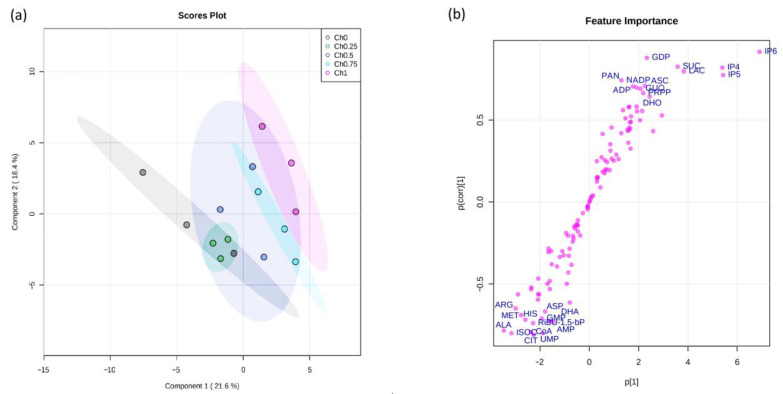
PCA and oPLS-DA score plots of tomato fruit metabolome from plants treated with different concentrations of chitosan (0, 0.25, 0.50, 0.75, and 1 mg mL^−1^). (**a**) PCA plots; (**b**) S-plot derived from oPLS-DA model between Ch0 and Ch1 groups.

Principal component analysis (PCA) showed that the first two principal components explain about the 40% of the variance in the data set (Figure 5a). Notably, the scores plot (Figure 5a) indicated that the first component, which represents 21.6% of the total variation, allowed us to distinguish the tomato groups according to chitosan concentration used for application. In addition, it showed that Ch0, Ch0.25, and Ch0.50 groups are clustered together, which indicates a similarity between them. However, the separation was only observed between the Ch0 group and either the Ch0.75 group or Ch1 group. As expected, this indicates that the high doses of chitosan applied to tomato plants have an effect on central carbon and energy metabolism in tomato fruits, in comparison to those of control.

To explain the variation between the Ch0 group and Ch1 group, the S-plot of the orthogonal partial least squares discriminant analysis (oPLS-DA) was generated (Figure 5b). The S-plot separates the variation in the data that arises from the chitosan treatment from other uncorrelated variation. Metabolites that scattered in the right-hand corner are positively correlated with the Ch1 group and include inositol phosphates (IP6, IP5, and IP4), sucrose (SUC), ascorbic acid (ASC), and lactic acid (LAC). By contrast, levels of TCA cycle substrates (i.e., citrate (CIT), isocitrate (ISOC)) and some of the amino acids (i.e., alanine (ALA), methionine (MET), arginine (ARG), histidine (HIS), aspartic acid (ASP)) appeared to be reduced in Ch1 treatment relative to the Ch0 control.

It is well-known that sugars, antioxidants, and minerals are essential in the human diet not only to avoid metabolic diseases but also to ensure metabolism regulation. Therefore, maintaining a high level of these components would be of great importance. The results of Table 3 show that Ch1 application to tomato shoots had a significant effect on the content of glucose, fructose, and sucrose, which were increased respectively by 27%, 28%, and 57%, compared to fruits from non-treated plants. These results confirm those found by metabolomics analysis. In addition, chitosan application did not show any effect on the content of α-tocopherol as well as on the levels of P and K; however, the level of N was increased by about 18%, 43%, and 38% in fruits from plants treated with Ch0.50, Ch0.75, and Ch1, respectively (Table 3).

Regarding pigments, all treatments resulted in an increase (Table 3). The application of Ch0.75 and Ch1 generated high levels of lycopene (84–90%) and flavonoids (39–51%).

## 4. Discussion

Presently, there is a worldwide trend to explore new alternatives to applying chemical treatments that reduce the loss of agricultural products. In fact, priority is given to methods that have no harmful side effects on human health and the environment. Among these methods, there is the use of chitosan, which has become a promising biostimulant and an adequate treatment for better development of fruits and vegetables. The effects of chitosan depend on many factors: (i) its applied dose, (ii) its application mode, (iii) its intrinsic properties (acetylation degree, molecular weight, pH, nitrogen content), and (iv) the environmental conditions of the plant (soil composition, temperature, water availability, salinity) [47,48,49,50,51].

Our data showed that foliar spray of chitosan, especially 0.75 mg mL^−1^, can improve the growth of tomato plants. This treatment increased leaf number, leaf area, and fresh and dry weights of shoots, in comparison to non-treated plants. Its application had, however, no effect on root growth. The improvement of vegetative growth under chitosan treatment might be attributed to a better functioning of chloroplasts and the increase in O_2_ production and CO_2_ fixation [52,53]. Therefore, to verify the effect of chitosan on photosynthetic performance, Fv/Fm and gs values were determined. Our data showed that the values of gs were reduced when non-treated plants pass one growth stage to another. However, these plants always kept the same values of Fv/Fm. Thus, the reduction in gs values might be dependent on soil water level and the age of plants [54,55]. Chitosan application affected Fv/Fm values differently. The values of Fv/Fm were increased after 2 weeks and 6 weeks of chitosan application. This result is in agreement with those found by Ahmad et al. [53,56]. The effect of chitosan could be due to the increase in the energy transfer efficiency of the antenna molecules which are localized in the photosystem II reaction center (PSII) [56] or the release of nitrogen from chitosan for use in the synthesis of the photosynthetic system components [57]. In addition, the improvement in chlorophyll fluorescence appears to be in good agreement with stomatal conductance aperture after the second and sixth week of treatment. The opening of stomata under chitosan application ensures strong assimilation of CO_2_ which, therefore, induces the photosynthetic activity of the plant. These results appear to be similar to those found by Sharma et al. [58]. However, after twelve weeks of applying treatment, a decrease in Fv/Fm values was observed only in plants treated with high doses of chitosan (0.75 and 1 mg mL^−1^). Czékus et al. [59] revealed that the application of 10 mg mL^−1^ chitosan to tomato plants can increase the emission of phytohormones, such as ethylene, by leaves, leading to leaf abscission. This study suggests that the decrease in quantum yields in leaves might be attributed to the increase in the ethylene content when plants received 6 mg mL^−1^ chitosan. In addition, the reducing effect of 1 mg mL^−1^ chitosan on plant and fruit growth might be related to the high production of ethylene [60]. Additionally, application of high doses of chitosan might result in a high availability of nitrogen, which is among the most important mineral elements that affect genes involved in ethylene biosynthesis [61].

Leaf and root damage caused by mechanical wounding trigger the synthesis of OPDA, JA, JA–Ile, and ABA. These phytohormones are accumulated in neighboring cells after the perception of damage-associated molecular patterns released from the damaged cells [62]. To ensure plant survival and growth–defense balance, the levels of JA and its bioactive form JA–Ile, as well as ABA, increase to induce the expression of wound-inducible genes, such as those encoding proteinase inhibitor II (*PI2*) and enzymes involved in the production of defense compounds, such as flavonoids and terpenoid indole alkaloids [31,63,64]. Similar impacts have been found for roots of *Arabidopsis thaliana* grown in substrate supplemented with chitosan (1 mg mL^−1^), leading to a higher accumulation of JA in comparison to plants grown without chitosan [20]. When leaves of tomato plants were treated with chitosan, especially with low and medium doses, an increase in the level of JA–Ile was visible in shoots only. After application of high doses of chitosan, however, the levels of JA–Ile in shoots were found to be unaltered. This might be due to a translocation of phytohormones to adjacent leaves or to the stem to be transported later to roots via the phloem [65]. Indeed, the contents of JA–Ile and ABA were induced in wounded roots when both treatments (Ch1 + wounding) were applied to shoots, suggesting their contribution in triggering systemic wound signals. To sort out the most effective applied dose of chitosan to heal shoot and root damage as well as to clarify whether the establishment of a systemic reaction is related to phytohormone translocation or linked to other signals triggered by chitosan action, a complex hormone profiling over different time periods might provide detailed information.

Our study has shown that chitosan application to shoots not only increased the above-ground biomass of plants but also affected the physico-chemical characteristics of fruits. The application of a medium dose of chitosan (Ch0.50) resulted in a significant increase in fruit yield and their longitudinal diameter; their shapes have become rounded instead of flattened. The application of a high dose of chitosan (Ch1), however, decreased fruit size and their relative water content, and increased Brix values and sugars contents. Fruit development is generally stimulated by three types of phytohormones: auxins, gibberellins, and cytokinins [66,67,68]. These phytohormones are produced inside the ovary after fertilization to stimulate cell division and expansion. It is tempting to speculate that the increase in the fruit size from plants treated with Ch0.50 might be due to an increase in the content of phytohormones, such as IAA. In contrast, the decrease in the fruit size from plants treated with Ch1 might be attributable to an early synthesis of ethylene, which is known to cause sugar accumulation and acidity reduction [59]. In addition, the application of chitosan to the aerial part of plants (leaves, stems, and fruits) increased the content of nitrogen in fruits. This increment might be attributed to releasing nitrogen from chitosan (polysaccharides—NH_2_) under the action of hydrolytic enzymes or promoting of xylem vessels’ growth which, therefore, increases nutrient uptake and nutritional status, especially nitrogen, to synthesize amino acids [24,69].

Fruit ripening is a complex process which is mainly characterized by the increase in sugars and aroma levels, the hydrolysis of starch and gross molecules, as well as the decrease in photorespiration [67]. The analysis of features of central carbon and energy metabolism showed a metabolic shift in the intermediates of glycolysis, TCA cycle, and the levels of metabolites involved therein, nucleotides, inositol phosphate, amino acids, and vitamin pathways in fruits after chitosan application. Our data showed that chitosan application to shoots affected the fruit metabolome by increasing the levels of sucrose and reducing the levels of organic acids, especially citrate. The generation of sucrose under chitosan application to the aerial part of plant can be due to the activation of amylase or the degradation of chitosan [70]. However, the decrease in citrate might be due to the involvement of the carbon skeletons resulting from glycolysis in other pathways, especially those of ascorbic acid and inositol phosphate. This is in agreement with the results obtained by Sajid et al. [71]. In addition, chitosan application has increased the levels of natural antioxidants (i.e., ascorbic acid, phytic acid, pantothenic acid, lycopene, and flavonoids). This could be attributed to a large production of sugars which were then converted to either glucose-6-phosphate or to different cellular metabolites to cope with the oxidative stress induced by chitosan treatment [39,72,73,74,75,76]. Thus, the increased levels of antioxidants in fruits of plants treated with chitosan when compared to untreated plants might point to the ability of chitosan to prevent cell damage.

## 5. Conclusions

Foliar application of chitosan exerts a beneficial impact on the aerial part of tomato plants, which is more pronounced in fruits than in leaves or roots. The analysis of fruits’ metabolites revealed that application of chitosan stimulated a series of primary and secondary metabolic pathways of carbon and nitrogen metabolisms. For instance, it enhanced CO_2_ fixation, nitrogen, and phosphorus levels, resulting in a higher production of sucrose, thereby providing carbon skeletons for synthesis of other metabolites, such as phospholipids, and antioxidants. In addition, chitosan applied to shoots or to roots led to a stronger plant response to wounding, as visible by higher levels of JA–Ile and ABA in roots after wounding of shoots. With these data, we showed that chitosan might be a very useful biostimulant in tomato cultivation and production.

## Figures and Tables

**Table 1:** Changes in transcript level of Allene Oxide Cyclase and 9-Cis-Epoxycarotenoid Dioxygenase in roots after foliar application of chitosan and wounding for 30 min. Means ± SD, *n* = 4. NS means non-significant differences between treatments while different letters indicate significant differences between treatments following two-way ANOVA (Duncan’s multiple range, *p* ≤ 0.05).

	Ch0		Ch0.25		Ch1	
	BW ^1^	AW ^1^	BW	AW	BW	AW
*AOC*	0.007 ± 0.004 ^a^	0.011 ± 0.009 ^a^	0.005 ± 0.003 ^a^	0.007 ± 0.005 ^a^	0.005 ± 0.002 ^a^	0.006 ± 0.003 ^a^
*NCED1*	0.012 ± 0.006 ^c^	0.053 ± 0.033 ^b^	0.010 ± 0.003 ^c^	0.055 ± 0.035 ^b^	0.025 ± 0.010 ^c^	0.084 ± 0.046 ^a^

^1^ BW, before wounding; AW, after wounding.

**Table 2:** Impact of foliar spray with chitosan on yield, fruit size, water content, Brix index, and pH.

Parameters	Ch0	Ch0.25	Ch0.50	Ch0.75	Ch1
**Total fruit weight (kg/plant) ^A^**	0.14 ± 0.02 ^c^	0.21 ± 0.01 ^a^	0.22 ± 0.01 ^a^	0.19 ± 0.01 ^ab^	0.18 ± 0.01 ^bc^
**Transverse diameter (mm) ^A^**					
**Min**	48.17 ± 5.52 ^a^	50.50 ± 2.29 ^a^	46.17 ± 1.61 ^a^	50.00 ± 0.87 ^a^	43.67 ±0.58 ^a^
**Max**	51.17 ± 5.39 ^a^	52.33 ± 3.75 ^a^	46.67 ± 1.26 ^ab^	51.17 ± 1.04 ^a^	43.67 ± 0.58 ^b^
**Longitudinal diameter (mm) ^A^**	40.33 ± 6.79 ^a^	47.50 ± 3.04 ^a^	44.67 ± 3.01 ^a^	44.17 ± 1.75 ^a^	41.00 ± 2.5 ^a^
**Shape index (SI = DL/DT) ^A^**					
**Min**	0.836 ± 0.01 ^b^	0.940 ± 0.04 ^ab^	0.970 ± 0.09 ^a^	0.884 ± 0.04 ^ab^	0.939 ± 0.06 ^ab^
**Max**	0.785 ± 0.06 ^b^	0.910 ± 0.08 ^ab^	0.959 ± 0.09 ^a^	0.864 ± 0.05 ^ab^	0.939 ± 0.06 ^a^
**Water content (%) ^B^**	93.50 ± 0.08 ^a^	93.87 ± 0.19 ^a^	93.28 ± 0.41 ^a^	93.47 ± 0.11 ^a^	91.77 ± 0.56 ^b^
**Total soluble sugar (°Brix) ^A^**	4.61 ± 0.87 ^b^	4.41 ± 0.70 ^b^	4.13 ± 0.07 ^b^	3.89 ± 0.49 ^b^	6.04 ± 0.54 ^a^
**pH ^A^**	4.55 ± 0.01 ^c^	4.58 ± 0.02 ^c^	4.59 ± 0.10 ^bc^	4.83 ± 0.13 ^a^	4.79 ± 0.13 ^ab^

Five treatments were applied until fruit ripening with 0 mg mL^−1^ (Ch0), 0.25 mg mL^−1^ (Ch0.25), 0.50 mg mL^−1^ (Ch0.50), 0.75 mg mL^−1^ (Ch0.75), and 1 mg mL^−1^ (Ch1) chitosan. ^A^ and ^B^ correspond to 3 and 5 replicates, respectively. Values followed by different lowercase letters are significantly different according to Duncan’s multiple range test (*p* ≤ 0.05).

**Table 3:** Chitosan effects on the levels of sugars, vitamin E, lycopene, and flavonoids as well as the acquisition of macronutrients.

Parameters	Ch0	Ch0.25	Ch0.50	Ch0.75	Ch1
**Glucose (mg g^−1^ FW) ^A^**	2.48 ± 0.06 ^b^	2.31 ± 0.14 ^b^	2.48 ± 0.17 ^b^	2.37 ± 0.05 ^b^	3.15 ± 0.36 ^a^
**Fructose (mg g^−1^ FW) ^A^**	4.18 ± 0.08 ^b^	3.94 ± 0.19 ^b^	4.13 ± 0.23 ^b^	4.10 ± 0.13 ^b^	5.35 ± 0.44 ^a^
**Sucrose (mg g^−1^ FW) ^A^**	4.76 ± 0.81 ^b^	4.58 ± 0.58 ^b^	5.15 ± 1.17 ^b^	4.85 ± 1.35 ^b^	7.49 ± 1.05 ^a^
**α-Tocopherol (µg g^−1^ DW) ^A^**	76.93 ± 21.64 ^a^	109.94 ± 31.37 ^a^	115.70 ± 41.88 ^a^	103.11 ± 13.18 ^a^	104.79 ± 25.29 ^a^
**Lycopene (mg kg^−1^ FW) ^B^**	16.40 ± 0.76 ^c^	24.73 ± 1.22 ^b^	25.67 ± 1.31 ^b^	30.20 ± 2.02 ^a^	31.18 ±1.38 ^a^
**Total flavonoids (mg g^−1^ DW) ^B^**	1.62 ± 0.11 ^d^	1.97 ± 0.22 ^c^	2.09 ± 0.09 ^bc^	2.44 ± 0.15 ^a^	2.25 ± 0.06 ^b^
**Macronutrients (mg g^−1^ DW) ^B^**					
**N**	25.29 ± 1.19 ^c^	26.31 ± 1.85 ^c^	29.89 ± 3.28 ^b^	36.25 ± 2.38 ^a^	34.89 ± 2.82 ^a^
**P**	5.57 ± 0.79 ^a^	6.66 ± 1.09 ^a^	6.47 ± 1.31 ^a^	6.58 ± 0.79 ^a^	6.70 ± 0.78 ^a^
**K**	26.99 ± 3.70 ^a^	26.71 ± 3.25 ^a^	29.16 ± 1.90 ^a^	29.93 ± 3.25 ^a^	28.42 ± 1.74 ^a^

Five treatments were applied until fruit ripening with 0 mg mL^−1^ (Ch0), 0.25 mg mL^−1^ (Ch0.25), 0.50 mg mL^−1^ (Ch0.50), 0.75 mg mL^−1^ (Ch0.75), and 1 mg mL^−1^ (Ch1) chitosan. ^A^ and ^B^ correspond to 3 and 5 replicates, respectively. Values followed by different lowercase letters are significantly different according to Duncan’s multiple range test (*p* ≤ 0.05).

## Data Availability

The data sets used in the current study are available from the corresponding author on request.

## Supplementary Materials

The following supporting information can be downloaded at: https://www.mdpi.com/article/10.3390/polym14225006/s1, Figure S1: Effect of foliar spray with chitosan on root biomass (**a**,**b**) of tomato plants. Five treatments were applied until fruit ripening with 0 mg mL^−1^ (Ch0), 0.25 mg mL^−1^ (Ch0.25), 0.50 mg mL^−1^ (Ch0.50), 0.75 mg mL^−1^ (Ch0.75), and 1 mg mL^−1^ (Ch1) chitosan, and four-month-old plants were analyzed. The results were expressed as the mean of 6 independent replicates ± SD. There were no significant differences between treatments following one-way ANOVA (Duncan’s multiple range, *p* ≤ 0.05).

## Abbreviations

ADP	adenosine diphosphate
ALA	alanine
AMP	adenosine monophosphate
ARG	arginine
ASC	ascorbate
ASP	aspartic acid
Ch	Chitosan
CIT	citrate
CoA	coenzyme A
DHA	dehydroascorbic acid
DHO	dihydroorotate
GDP	guanosine diphosphate
GMP	guanosine monophosphate
GUO	guanosine
HIS	histidine
IP4	sum of inositol tetrakisphosphates
IP5	sum of inositol pentakisphosphates
IP6	inositol hexakisphosphate
ISOC	isocitrate
LAC	lactate
MET	methionine
NADP	nicotinamide adenine dinucleotide phosphate (oxidized)
PAN	pantothenate
PRPP	phosphoribosyl pyrophosphate
SUC	sucrose
RiBU-1,5-bP	ribulose1,5-bisphosphate
UMP	uridine monophosphate
