# Supplementary material for: The Effect of Chitosan on Plant Physiology, Wound Response, and Fruit Quality of Tomato
Source: Polymers (Basel). 2022 Nov 18;14(22):5006. doi: 10.3390/polym14225006 (PMC9692869; doi:10.3390/polym14225006)
Supplement: Supplementary file 1 [file polymers-14-05006-s001.zip › polymers-1960664-supplementary.pdf]

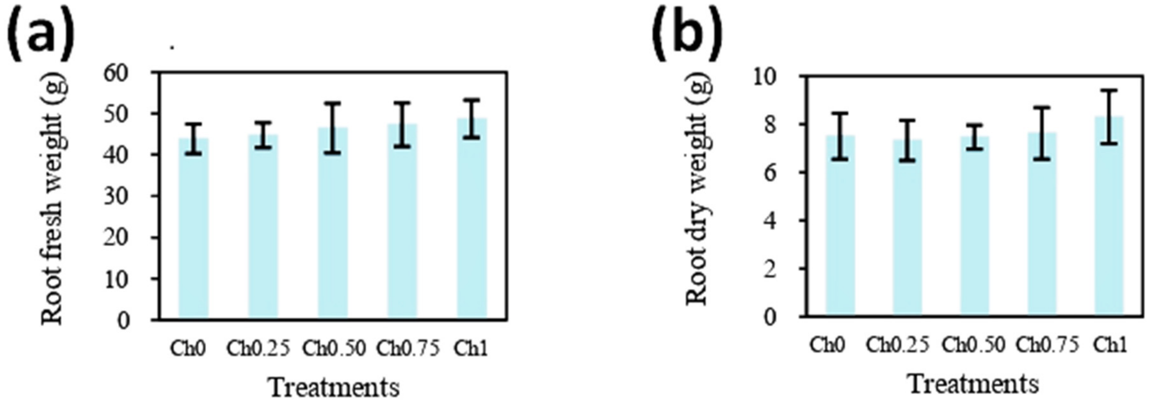

Figure S1. Effect of foliar spray with chitosan on root biomass (**a**,**b**) of tomato plants. Five treatments were applied until fruit ripening with 0 mg mL^{-1} (Ch0), 0.25 mg mL^{-1} (Ch0.25), 0.50 mg mL^{-1} (Ch0.50), 0.75 mg mL^{-1} (Ch0.75), and 1 mg mL^{-1} (Ch1) chitosan, and four-month-old plants were analyzed. The results were expressed as the mean of 6 independent replicates ± SD. There were no significant differences between treatments following one-way ANOVA (Duncan's multiple range, $p \leq 0.05$).
